# Comparative evaluation of radiological anatomy knowledge and accuracy of ChatGPT‐5, Gemini 2.5, and Grok 4 across normal and thinking modes

**DOI:** 10.1002/ase.70292

**Published:** 2026-06-26

**Authors:** Ismail Sivri, Furkan Mehmet Ozden, Halit Celik, Ozgur Gokturk, Tuncay Colak

**Affiliations:** ^1^ Department of Anatomy, Faculty of Medicine Kocaeli University İzmit Türkiye; ^2^ Department of Anatomy, Institute of Health Sciences Kocaeli University İzmit Türkiye

**Keywords:** artificial intelligence, diagnostic imaging, large language models, medical education, radiologic anatomy

## Abstract

This study compared the performance of three large language models, ChatGPT‐5 Plus, Gemini 2.5 Pro, and SuperGrok 4, in identifying anatomical structures on radiographic images using standardized anatomical terminology. Thirty radiographs from different body regions were selected from an open‐access atlas and analyzed by the models in Normal and Thinking modes using standardized prompts based on Terminologia Anatomica (version 2.07). Responses were evaluated independently by two anatomists using a 0–2 scoring system. Overall accuracy across both modes and models ranged from 47.4% to 85.7%. Data were analyzed using Friedman and Wilcoxon signed‐rank tests. Temporal response consistency was assessed with weighted kappa coefficients. Gemini 2.5 Pro and ChatGPT‐5 Plus significantly outperformed SuperGrok 4 in both modes. In Normal mode, Gemini 2.5 Pro achieved the highest overall accuracy (82.7%), significantly exceeding ChatGPT‐5 Plus (60.7%, *p* = 0.001) and SuperGrok 4 (47.4%, *p* < 0.001). In Thinking mode, accuracies were 85.7% for Gemini 2.5 Pro, 77.6% for ChatGPT‐5 Plus, and 49.5% for SuperGrok 4. Gemini 2.5 Pro demonstrated a significant advantage over ChatGPT‐5 Plus only in Normal mode (*p* = 0.001), whereas Thinking mode significantly improved performance only for ChatGPT‐5 Plus (*p* = 0.01). Temporal stability analysis showed high response consistency for Gemini 2.5 Pro and SuperGrok 4 across all modes (*r* > 0.94, *p* < 0.001). Conversely, ChatGPT‐5 Plus' stability decreased from substantial agreement in normal mode (*r* = 0.697, *p* < 0.001) to moderate agreement in Thinking mode (*r* = 0.539, *p* < 0.001). Despite their educational potential, these models need refinement to reliably identify anatomical structures on radiographic images.

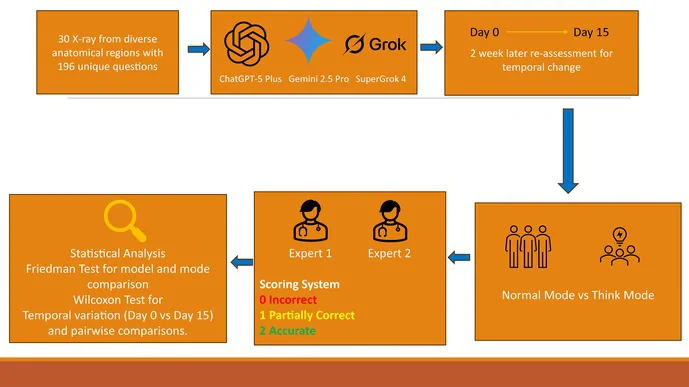

## Graphical abstract

Workflow for comparing three LLMs in radiographic anatomical identification using 30 radiographs and 196 anatomy questions across Normal and Thinking modes, with expert scoring and temporal consistency analysis.

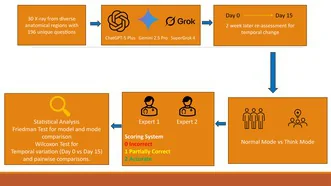

## INTRODUCTION

Since its discovery in 1895, conventional radiography has remained the primary imaging modality for the initial assessment of musculoskeletal and rheumatic disorders, making it indispensable for clinicians.^1^ For most medical students, developing proficiency in identifying structures on radiographs and correlating these findings with anatomy is a fundamental requirement for their future clinical practice.^2^ As generative artificial intelligence (GenAI) becomes increasingly integrated into medical education, resources for learning radiography are rapidly evolving. With large language models (LLMs) now able to identify anatomical structures across various imaging modalities (e.g., conventional radiographs, CT, and MRI), ongoing accuracy benchmarking is crucial for determining when LLMs are ready for reliable educational use.^3^ Older LLMs have not yet achieved reliable accuracy for use in anatomy education settings.^3^ However, the ability of reasoning‐enhanced LLMs to interpret radiological anatomy across body regions remains underexplored.

While prior research has demonstrated that LLMs perform well on text‐based anatomy questions,^4^, ^5^, ^6^, ^7^ their accuracy declines substantially when interpreting medical images.^8^, ^9^, ^10^, ^11^ For example, one study systematically tested the visual recognition performance of four prominent LLMs, including GPT‐4o, Claude, Gemini, and Copilot, on 144 histological images from multiple tissue types. The models achieved over 90% accuracy on text‐based medical questions, but their ability to interpret histological images was significantly lower, ranging from 50.7% to 80.1% across models, with Gemini achieving the highest accuracy.^8^ In a comparative study of GPT‐4 and GPT‐4o on Japanese Surgical Board Exam questions, GPT‐4o achieved 83% on text‐only questions but dropped to 67% with images, and adding visual data did not significantly improve overall results.^9^ Additional studies have confirmed this pattern across medical disciplines, including radiology,^12^, ^13^ pathology,^8^ and anatomy,^11^, ^14^ consistently showing that visual interpretation lags behind text‐based reasoning.

Existing studies have primarily evaluated earlier model versions (e.g., GPT‐4, Gemini 1.5) using small or non‐standardized image sets.^14^, ^15^ Furthermore, whether “Thinking modes” improve visual anatomical identification has not been systematically investigated. A critical gap exists in understanding whether the latest reasoning‐optimized LLMs (i.e., ChatGPT‐5, Gemini 2.5, and Grok 4) have reached sufficient accuracy for reliable use in radiological anatomy education settings.

Evidence‐based guidance is crucial for integrating AI tools into anatomy education, as unreliable models may propagate misconceptions when used unsupervised. Therefore, this study investigates the practical readiness and compares the accuracy of ChatGPT‐5 Plus, Gemini 2.5 Pro, and SuperGrok 4 in identifying radiographic anatomical structures.

## METHODS

The study analyzed 30 radiographs from the open‐access ClinicalAnatomy.ca radiological atlas across nine anatomical regions using 196 specific anatomical questions. The dataset ranged in complexity from simple chest views to complex structures such as the wrist and shoulder^16^ (see File S1 for detailed Image Preparation and Control Measures). Ethical approval was exempt as the study did not involve human subjects.

### Prompting strategies: Normal versus thinking mode

**FIGURE 1:**
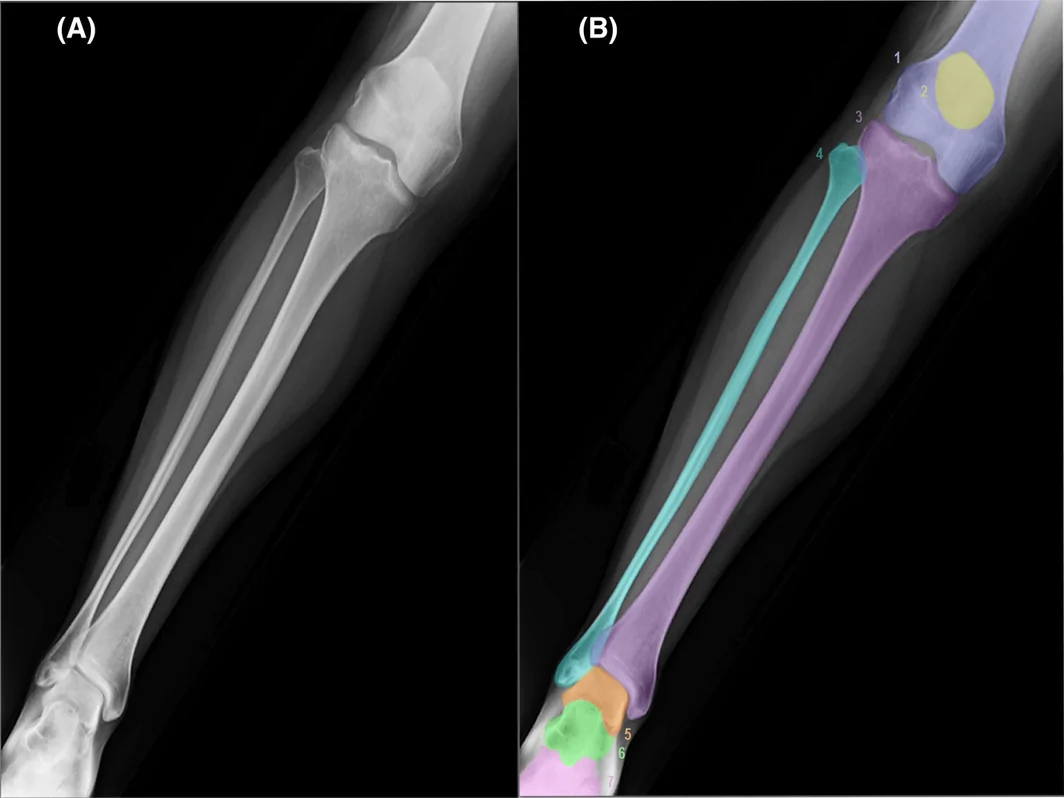
The non‐colored (A), colored, and custom‐numbered (B) X‐ray images.

Each image pair (Figure 1) (original and colored with custom numbering) was processed using two prompting strategies:

#### Normal mode

A standard “zero‐shot” approach evaluating the models' intuitive pattern recognition and rapid identification based on baseline training.

#### Thinking mode

Leverages Chain‐of‐Thought (CoT) reasoning to generate intermediate logical steps, facilitating the deliberate, step‐by‐step spatial analysis needed for complex radiological landmarks and overlapping structures (see File S2 for ChatGPT Thinking Mode).

### LLM selection, response collection, and evaluation

Premium versions of ChatGPT‐5 Plus, Gemini 2.5 Pro, and SuperGrok 4 were evaluated using a standardized prompt. To eliminate residual context and ensure an unbiased evaluation, “Memory” and “Chat History” features were disabled, and each image pair was analyzed in a new, independent chat session.^17^, ^18^, ^19^ The entire procedure was repeated two weeks later under identical conditions to assess consistency and temporal reliability.

A panel of five anatomists determined the prompts for the Normal and Thinking Modes (see File S1 for prompts). These prompts required the LLM to map the radiographs' expert‐defined numerical identifiers to their corresponding Terminologia Anatomica nomenclature.

To ensure data standardization and consistency across AI architectures, all LLM outputs were restricted to JavaScript Object Notation (JSON) format. This structured, machine‐readable key‐value template mitigated distortions caused by unstructured narratives, hallucinations, and conversational filler by requiring models to adhere strictly to a predefined output format.^20^

Five expert anatomists distributed and uploaded the questions to the LLMs. Two anatomists independently evaluated each response using a 3‐point scale (0: incorrect, 1: partially correct/superimposed structures, 2: accurate). Disagreements were resolved by a third anatomist. All LLM responses were assessed against the anatomical nomenclature of the FIPAT Terminologia Anatomica (version 2.07).^21^

### Qualitative error pattern assessment

Incorrect model responses were qualitatively reviewed by two independent anatomists to identify recurrent, systematic error patterns rather than isolated inaccuracies. This qualitative analysis was essential because quantitative accuracy scores alone cannot distinguish random errors from systematic biases, which carry important educational implications. The anatomists evaluated errors relative to the target anatomical structure and image context, paying close attention to complex or superimposed regions and categorizing error types (such as sequence‐based naming, superimpositions, and laterality errors), resolving any discrepancies through consensus discussion. Finally, the frequencies of these systematic patterns were estimated and classified as either model‐specific or common across multiple models and modes.

### Statistical analysis

An a priori power analysis (Wilcoxon signed‐rank framework, *dz* = 0.5, *α* = 0.05, one‐tailed, desired power = 0.95) indicated a minimum requirement of 47 paired observations. The study's final sample size (*n* = 196 items) yielded an achieved power >0.99. Power analyses were performed using G*Power version 3.1.9.7.

All statistical analyses were conducted using IBM SPSS Statistics (version 31), with categorical data summarized via frequencies (*n*) and percentages (%). The regional accuracy heatmap was generated using R (version 4.6.0). Within‐mode and cross‐mode LLM comparisons were analyzed using the Friedman test, followed by post hoc pairwise Wilcoxon signed‐rank tests where significant. A two‐tailed *p* < 0.05 denoted statistical significance.

The Wilcoxon test effect size (*r*) was calculated as follows:

$$r=|Z|/\sqrt{N},$$

where |*Z*| is the absolute standardized test statistic and *N* is the total observations (*N* = 196). Effect sizes were classified as small (0.1 ≤ *r* < 0.3), medium (0.3 ≤ *r* < 0.5), or large (*r* ≥ 0.5).

Temporal reliability over a 2‐week interval was assessed using Cohen's weighted Kappa (wK) to account for the ordinal scale (0, 1, 2). Agreement levels were interpreted using Landis and Koch benchmarks: slight (0.00–0.20), fair (0.21–0.40), moderate (0.41–0.60), substantial (0.61–0.80), and almost perfect (0.81–1.00).^22^

## RESULTS

**FIGURE 2:**
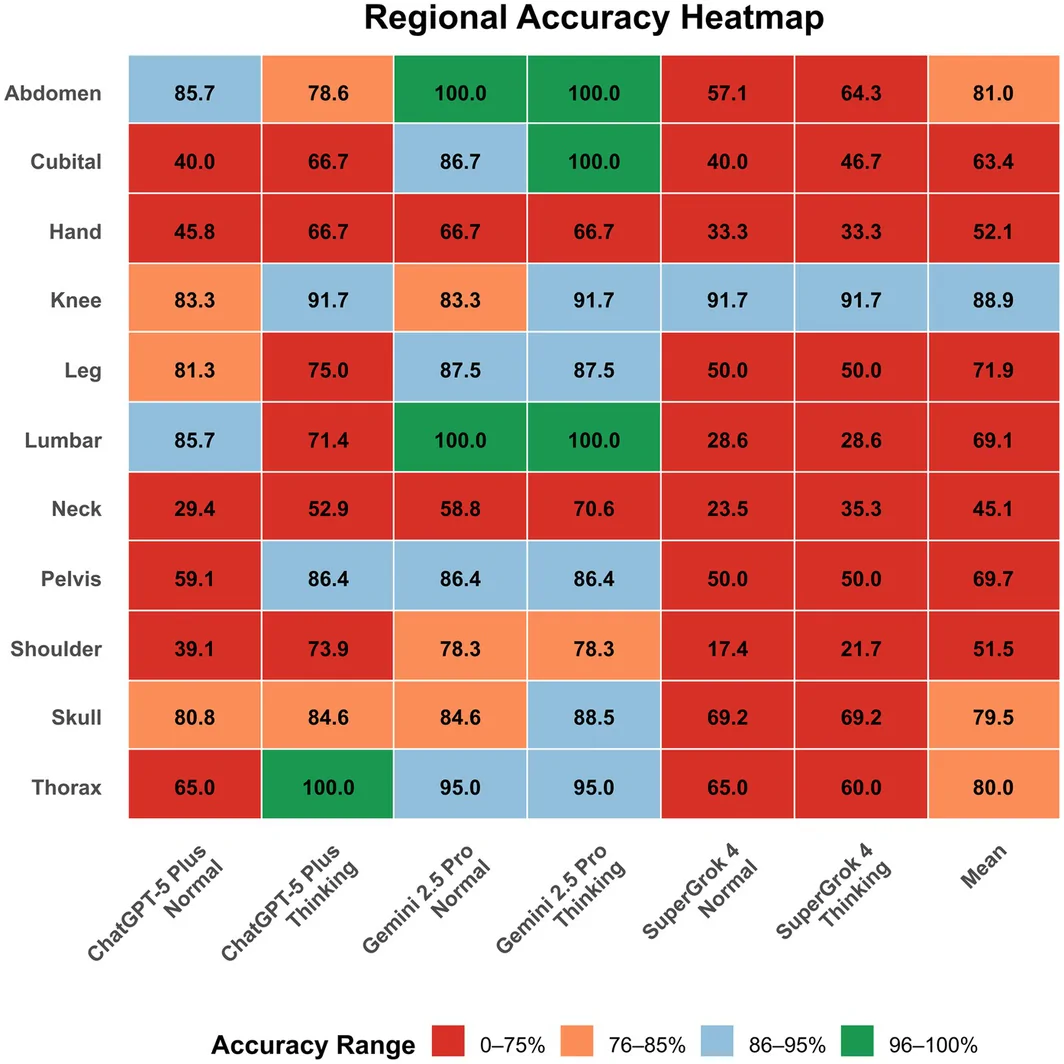
Heatmap showing the comparative accuracy of ChatGPT‐5 Plus, Gemini 2.5 Pro, and SuperGrok 4 across anatomical regions in Normal and Thinking modes. Color coding was based on predefined accuracy thresholds: red (0%–75%), orange (76%–85%), blue (86%–95%), and green (96%–100%).

Accuracy outcomes are presented in Figure 2. Gemini 2.5 Pro achieved the highest overall accuracy in both Normal (82.7%) and Thinking (85.7%) modes, followed by ChatGPT‐5 Plus (60.7% and 77.6%) and SuperGrok 4 (47.4% and 49.5%). The Thinking mode improved ChatGPT‐5 Plus' performance by 16.9 percentage points, while improvements for Gemini 2.5 Pro (+3.0%) and SuperGrok 4 (+2.1%) were minimal. Regionally, all models performed worst in the neck (*μ* = 45.1%), shoulder (*μ* = 51.5%), and hand (*μ* = 52.1%) regions, and best in the knee (*μ* = 88.9%), abdomen (*μ* = 81.0%), and thorax (*μ* = 80.0%; Figure 2).

Gemini 2.5 Pro's accuracy in normal mode (82.7%) significantly outperformed ChatGPT‐5 Plus (60.7%, *p* = 0.001, *r* = 0.24) and SuperGrok 4 (47.4%, *p* = 0.000, *r* = 0.41, Table 1). ChatGPT‐5 Plus also surpassed SuperGrok 4 in this mode (*p* = 0.015, *r* = 0.17). In Thinking mode, both Gemini 2.5 Pro (85.7%, *p* = 0.000, *r* = 0.43) and ChatGPT‐5 Plus (77.6%, *p* = 0.001, *r* = 0.34) maintained a statistically significant superiority over SuperGrok 4 (49.5%). Notably, the difference between Gemini 2.5 Pro and ChatGPT‐5 Plus was not statistically significant in Thinking mode (*p* = 0.209). This finding was verified through re‐analysis of the raw data, confirming the p‐value and effect size calculations. The transition from Normal to Thinking mode yielded a statistically significant increase in accuracy for ChatGPT‐5 Plus (*p* = 0.010, *r* = 0.18). No significant performance changes were observed for Gemini 2.5 Pro (*p* = 0.599) or SuperGrok 4 (*p* = 0.756) when switching to the Thinking mode.

**TABLE 1:** Inter‐model and within‐model statistical comparisons of identification accuracy across Normal and Thinking modes (*Z* values based on the Wilcoxon signed‐rank test).

Category	Pairwise comparison (with accuracy %)	*p* value (sig.)	*Z* value	Effect size (*r*)
Between‐model (normal mode)	Gemini 2.5 Pro (82.7%) vs. ChatGPT‐5 Plus (60.7%)	0.001	−3.294	0.24
	ChatGPT‐5 Plus (60.7%) vs. SuperGrok 4 (47.4%)	0.015	2.443	0.17
	Gemini (82.7%) vs. SuperGrok 4 (47.4%)	0.000	5.737	0.41
Between‐model (thinking mode)	Gemini 2.5 Pro (85.7%) vs. ChatGPT‐5 Plus (77.6%)	0.209	−1.255	0.09
	ChatGPT‐5 Plus (77.6%) vs. SuperGrok 4 (49.5%)	0.001	4.698	0.34
	Gemini 2.5 Pro (85.7%) vs. SuperGrok 4 (49.5%)	0.000	5.953	0.43
Within‐model (mode impact)	ChatGPT‐5 Plus Thinking (77.6%) vs. Normal (60.7%)	0.01	−2.565	0.18
	Gemini 2.5 Pro Thinking (85.7%) vs. Normal (82.7%)	0.599	−0.526	0.04
	SuperGrok 4 Thinking (49.5%) vs. Normal (47.4%)	0.756	−0.310	0.02

The evaluation of temporal stability after a two‐week interval revealed that Gemini 2.5 Pro and SuperGrok 4 achieved excellent agreement (test–retest reliability) across both modes, with weighted kappa values (wK) exceeding 0.94 (Table 2). While ChatGPT‐5 Plus showed substantial agreement in Normal mode (wK = 0.697, *p* < 0.001), moderate agreement was observed in the model's Thinking mode (wK = 0.539, *p* < 0.001).

**TABLE 2:** Intra‐model and intra‐mode reliability of LLM responses (test–retest reliability) was evaluated via Cohen's weighted kappa across a two‐week interval.

Model	Mode	Weighted kappa (wK)	*p* value (sig.)
ChatGPT‐5 Plus	Normal	0.697	0.000
	Thinking	0.539	0.000
Gemini 2.5 Pro	Normal	0.947	0.000
	Thinking	0.975	0.000
SuperGrok 4	Normal	0.959	0.000
	Thinking	0.948	0.000

### Qualitative error patterns

Qualitative analysis of 347 incorrect responses revealed recurrent error patterns in complex anatomical regions. In the wrist, models often listed the carpal bones in a standard anatomical sequence rather than identifying the target structure; for example, when the correct answers were os trapezoideum and os capitatum, ChatGPT‐5 Plus in Normal mode responded with os scaphoideum and os lunatum, which is consistent with the proximal carpal row. This sequence‐based error occurred in approximately 16.6% (4/24) of hand/wrist questions across models. In superimposed regions, such as the paranasal sinuses, models tended to name the overlying bone, such as the frontal bone, rather than the sinus itself, such as the sinus frontalis. This “overlying structure preference” accounted for approximately 19.2% (5/26) of incorrect skull and facial bone responses. Thinking mode reduced sequence‐based errors in ChatGPT‐5 Plus by approximately 40% but had a minimal effect on SuperGrok 4. These findings suggest that some errors reflected systematic tendencies, such as “safe guesses” of familiar structures, rather than random misidentifications.

## DISCUSSION

Benchmarking three LLMs on 30 radiographs (196 identification tasks) showed Gemini 2.5 Pro attained the highest accuracy (82.7% in Normal mode; 85.7% in Thinking mode), outperforming ChatGPT‐5 Plus (60.7%; 77.6%) and SuperGrok 4 (47.4%; 49.5%). Thinking mode markedly improved ChatGPT‐5 Plus (+16.9% points) but not the others. Temporal consistency was excellent for Gemini and SuperGrok (wK > 0.94) and only moderate–substantial for ChatGPT‐5 Plus (wK 0.539–0.697). Regionally, models struggled most with neck, shoulder, and hand, and performed best on knee, abdomen, and thorax.

### Comparison with prior work and contribution to the literature

Our findings demonstrate substantial generational progress relative to prior benchmarks, such as the New England Journal of Medicine Image Challenge, in which GPT‐4o achieved only 59.6% accuracy. Gemini 2.5 Pro (82.7%) and ChatGPT‐5 Plus in Thinking mode (77.6%) significantly exceed these earlier baselines, reflecting improved autonomous visual interpretation.^12^ However, consistent with Suh et al.,^12^ our models still performed best on anatomically distinct regions (e.g., knee, abdomen) and worst on regions requiring the integration of superimposed structures (e.g., neck, shoulder), suggesting that true spatial reasoning remains incomplete.

In another study, six multimodal LLMs were compared on 56 complex neuroradiology cases from JAMA Neurology and JAMA. Claude 3.5 achieved the highest accuracy (80.4%) with combined image and text input. However, their independent diagnostic capabilities for image‐only tasks remained notably limited, with accuracy in identifying pathologic locations ranging from 21.5% to 63.1%.^13^ Our study similarly found that LLMs struggle with pure image interpretation, but the higher baseline accuracies we observed (47.4%–85.7%) suggest improvement over earlier models. The persistent difficulty with the neck (mean accuracy 29.4%–70.6% across models) and shoulder (17.4%–78.3%) mirrors the neuroradiology finding that complex, superimposed regions remain challenging.^13^ These findings suggest that current multimodal LLMs may still rely significantly on textual cues, which can occasionally overshadow autonomous image interpretation. Consequently, further refinements in radiologic image analysis could enhance reliability and better support its integration into clinical diagnostic workflows.

### Systematic error patterns

In a recent study, it was observed that LLMs often follow a “safe guess” pattern, selecting well‐known anatomical structures rather than identifying the specific targets shown in the image. The models struggle with depth and orientation in two‐dimensional views, often focusing on prominent visual cues rather than interpreting complex spatial relationships in anatomy.^23^ Our qualitative error analysis supports this pattern. Systematic errors occurred in over 70% of incorrect responses in the wrist and paranasal sinus regions across all three models, most prominently in SuperGrok 4. Thinking mode reduced this “sequential guessing” error in ChatGPT‐5 Plus but had little effect on the other models. End users should therefore recognize that LLMs may produce confident but incorrect responses following predictable patterns.

### Regional variability

Regional variability in our study mirrors prior evidence that LLM performance in radiology and anatomy is highly task‐ and domain‐dependent. Ueda et al.^15^ showed that ChatGPT‐4 performed much better in cardiovascular than musculoskeletal radiology (79% vs. 42%), and another quiz‐based radiology study likewise found wide subspecialty variation, with the highest accuracy in cardiovascular, head and neck, and pediatric radiology, but lowest in musculoskeletal imaging.^15^ A cadaveric anatomy study reported a similar pattern, in which GPT‐4o correctly identified only 22.26% of labeled structures overall, performing far better on osteological structures than on thoracic organs.^11^ Our results extend these region‐specific findings to the identification of radiographic anatomy. Gemini 2.5 Pro performed best overall, achieving 100% accuracy in the abdomen and lumbar regions, whereas ChatGPT‐5 Plus was strongest in the thorax and knee, and SuperGrok 4 in the knee region. Across models, performance was weakest in complex regions such as the neck, shoulder, and hand, suggesting that LLM accuracy varies by anatomical region and is influenced by complexity, superimposition, and the availability of contextual visual information.

### Model comparison and evolution

Prior studies show shifting model rankings. ChatGPT achieved 100% on 23 anatomy questions (knowledge‐based, USMLE Step 1, and radiographs),^14^ yet a more recent brain MRI study found that Gemini outperformed Grok, which in turn outperformed GPT‐4.^24^ Our results reflect this evolving landscape, in which Gemini 2.5 Pro outperformed ChatGPT‐5 Plus in Normal mode (82.7% vs. 60.7%, *p* = 0.001), though the gap narrowed to non‐significance in Thinking mode (85.7% vs. 77.6%, *p* = 0.209), suggesting that rankings depend heavily on task type and reasoning configuration. Notably, while earlier versions of Gemini failed to recognize medical images,^25^, ^26^ our findings confirm substantial improvement, consistent with recent evaluations showing that Gemini can outperform ChatGPT on medical image‐based tasks.^8^, ^27^ Given accuracy ranges of 47.4%–85.7% across models and modes, LLMs are best positioned as supplementary self‐assessment tools rather than autonomous graders or tutors, provided that expert anatomists review AI‐generated answers. Even the highest observed accuracy (Gemini 2.5 Pro Thinking: 85.7%) falls below the thresholds expected for clinical decision support,^10^, ^28^, ^29^ precluding autonomous clinical application.

CoT prompting has previously improved LLM accuracy on oral and maxillofacial surgery questions^26^ and bilingual nuclear medicine examinations.^30^ In this study, ChatGPT‐5 Plus's Thinking mode significantly improved identification of radiographic anatomy, whereas Gemini 2.5 Pro and SuperGrok 4 showed only small, non‐significant gains. This suggests that the benefits of CoT‐style reasoning are model‐specific, likely reflecting differences in architecture and training.

### Temporal consistency

Previous studies show that LLM accuracy and reliability vary substantially by task format, modality, and model. In text‐based medical science assessments, performance is often high. For example, GPT‐4o and Claude achieved 89.7% and 87.5% accuracy on embryology USMLE‐style questions, with good test–retest consistency for GPT‐4o, Claude, and Gemini.^5^ Similarly, five LLMs averaged 91.1% accuracy on histology MCQs, although reliability varied by model, ranging from Claude (ICC = 0.931) to Gemini (ICC = 0.562).^6^ In gross anatomy, ChatGPT‐4 similarly outperformed other models on upper‐limb MCQs and generated clinically oriented curriculum questions, but still required human oversight.^31^ However, image‐based tasks appear less stable. In unlabeled anatomical images, ChatGPT‐4o reached only 68.0% factual accuracy but showed better temporal consistency than Claude 3.5 Sonnet (ICC = 0.770 vs. 0.505), suggesting lower reliability for visual interpretation than text‐only evaluations.^3^ Our radiographic anatomy results similarly showed model‐dependent performance. Gemini 2.5 Pro had the highest Normal‐mode accuracy (82.7%), exceeding ChatGPT‐5 Plus (60.7%) and SuperGrok 4 (47.4%), while Gemini 2.5 Pro and SuperGrok 4 showed excellent temporal stability (wK > 0.94). ChatGPT‐5 Plus was less reliable, declining from substantial agreement in Normal mode (wK = 0.697) to moderate agreement in Thinking mode (wK = 0.539), indicating that reasoning mode may improve accuracy at the cost of greater response variability. Together with recent internal medicine board‐question findings showing Gemini 2.5 Flash outperforming ChatGPT‐5 and ChatGPT‐4o on text‐based tasks,^32^ these suggest that LLM performance depends on task type, modality, model version, and the prioritization of accuracy versus consistency.

### Practical educational implications

Based on our findings, we offer the following practical recommendations for medical educators considering LLM integration into radiological anatomy instruction:

1. Align model selection with performance characteristics: For X‐ray radiological anatomy tasks, Gemini 2.5 Pro showed the most balanced profile, combining high accuracy (82.7%–85.7%) with strong stability (wK = 0.947–0.975). ChatGPT‐5 Plus benefited substantially from reasoning mode (60.7%–77.6%) but showed lower session‐to‐session consistency (wK = 0.539–0.697). SuperGrok 4 was less suitable for this instructional context because of its limited accuracy (47.4%–49.5%).
2. Avoid LLM‐dependent assessment in complex regions: Educators should not rely on LLMs for grading or tutoring in anatomically complex areas.
3. Explicitly teach error recognition: The systematic error patterns we identified (sequence‐based naming, overlying structure preference) should be demonstrated to students so they can critically evaluate LLM outputs.
4. Use LLMs as supplementary, not primary, resources: Given their current overall accuracy ranges, LLMs are not yet reliable as autonomous tutors. Expert oversight remains essential for image interpretation.
5. Consider model‐specific strengths: ChatGPT‐5 Plus excelled in the thorax (up to 100%); Gemini 2.5 Pro excelled in the abdomen and lumbar spine (100%); SuperGrok 4 excelled in the knee (91.7%). Educators might match the model to the region based on these strengths.

## LIMITATIONS

The first limitation is that the assessment methodology was affected by the hierarchical granularity of anatomical nomenclature and prompt ambiguity. For instance, when a specific structure like the frontal sinus was demarcated, some models returned the broader parent term, frontal bone. Since the prompt requested the “anatomical structure,” models might have struggled to distinguish between the specific air cavity and the surrounding osseous structure. To maintain rigorous diagnostic standards and avoid overestimating performance, such non‐specific identifications received only partial credit, potentially underestimating the models' true recognition ability within a broader topographical context.

Second, the unequal distribution of images and structures across regions may have overrepresented certain areas, limiting a balanced assessment of overall anatomical proficiency.

Third, the image‐labeling strategy may have influenced performance. Although color‐coded numbered labels reduced ambiguity in adjacent areas, superimposed structures remained difficult to control. Future studies should systematically compare different labeling methods, including arrows, outlines, lines, and numbers, to determine their precise effect on model accuracy.

## CONCLUSION

This study showed that LLMs can identify anatomical structures in radiographic images, although accuracy varied across models and reasoning modes. Overall accuracy ranged from 47.4% to 85.7%. Gemini 2.5 Pro achieved the highest accuracy (82.7% in Normal mode and 85.7% in Thinking mode), followed by ChatGPT‐5 Plus (60.7% and 77.6%, respectively), which showed significant improvement with reasoning enhancement (*p* = 0.01). SuperGrok 4 showed the lowest accuracy (47.4% and 49.5%, respectively). Reassessing these latest model versions provides insight into their evolving diagnostic and educational potential. The findings suggest that reasoning‐enhanced modes may improve the interpretive reliability of LLMs in radiological anatomy, particularly for ChatGPT‐5 Plus. However, given the remaining gap from expert‐level performance, these tools should be used as supplementary resources under expert oversight rather than as independent tutors or graders.

## AUTHOR CONTRIBUTIONS

**Furkan Mehmet Ozden:** Validation; methodology; investigation; writing – review and editing. **Tuncay Colak:** Supervision; project administration. **Halit Celik:** Investigation; validation. **Ozgur Gokturk:** Investigation; validation. **Ismail Sivri:** Writing – original draft; methodology; conceptualization; writing – review and editing; formal analysis; investigation.

## FUNDING INFORMATION

The authors received no financial support for the research, and authorship. The publication of this article was financially supported by Kocaeli University.

## CONFLICT OF INTEREST STATEMENT

The authors declare that they have no conflicts of interest.

## ETHICS STATEMENT

According to the decision of the Kocaeli University Non‐Interventional Clinical Research Ethics Committee (Approval Code: GOKAEK‐2025/21/35), this study did not require formal ethical approval because it involved no human participants, identifiable data, or biological materials and used exclusively open‐access, anonymized radiological image sources.

## Supporting information


File S1.

File S2.

## Data Availability

The X‐ray images used in this study were obtained from the open‐access radiological atlas available at https://www.clinicalanatomy.ca/radiology.html. Evaluation files and model response datasets can be provided by the corresponding author upon reasonable request.
