# Supplementary material for: Comparative evaluation of radiological anatomy knowledge and accuracy of ChatGPT‐5, Gemini 2.5, and Grok 4 across normal and thinking modes
Source: Anat Sci Educ. 2026 Jun 26;19(9):1377–85. doi: 10.1002/ase.70292 (PMC13545335; doi:10.1002/ase.70292)
Supplement: Supplementary file 1 [file ASE-19-1377-s001.docx]

**Supplementary material for:**

**Comparative Evaluation of Radiological Anatomy Knowledge and Accuracy of ChatGPT-5, Gemini 2.5, and Grok 4 Across Normal and Thinking Modes**

**Supplementary Methods: Image Preparation, Control Measures, and Prompt Protocol**

**Image Preparation and Control Measures**

The images were sourced from the open-access radiological atlas provided by clinicalanatomy.ca, curated by the University of British Columbia. They were downloaded directly from Clinicalanatomy.ca at their original resolution to prevent quality loss[1]. To address structural difficulty, two anatomists independently evaluated the images. They identified anatomical structures through a consensus-based approach, ensuring that only mutually agreed-upon landmarks were numbered for the analysis.

To ensure the models relied on visual processing rather than simple data retrieval, each anatomical region of interest was manually labeled and numbered using Microsoft Paint (Microsoft Corp., Redmond, WA). Each image was saved in the Bitmap (BMP) format without compression to preserve clarity and ensure an accurate depiction of anatomical structures. Although the base images are publicly available, the specific numbering was uniquely generated for this study. Consequently, even with active internet access, the models could not simply "look up" the answers. They had to use their built-in multimodal (vision) capabilities to generate correct answers for anatomical structures assigned a corresponding number. Furthermore, to maintain a standardized and equitable testing environment, internet access was kept enabled for all three models (ChatGPT-5 Plus, Gemini 2.5 Pro, and SuperGrok 4), as this feature is integral and cannot be disabled in Gemini 2.5 Pro and SuperGrok 4.

| Mode | Prompt |
| --- | --- |
| Normal mode | Act like an anatomist. I will give you both a normal radiological image and a version where certain regions are colored and numbered. Your tasks: 1) Identify which anatomical region the image represents. 2) List the names of the colored anatomical structures in numerical order according to Terminologia Anatomica. 3) Provide only the answer in JSON format. (JSON format: {"region": "Anatomical region name", "structures": [{"number": 1, "name": "Structure name"}, {"number": 2, "name": "Structure name"}]}) |
| Thinking mode | Act like an anatomist. I will give you both a normal radiological image and a version where certain regions are colored and numbered. Your tasks: 1) Step by step, think about which anatomical region the image might represent. 2) For each numbered area, identify the anatomical structure according to Terminologia Anatomica. 3) After completing your reasoning, provide only the final answer in JSON format (do not include your reasoning steps). (JSON format: {"region": "Anatomical region name", "structures": [{"number": 1, "name": "Structure name"}, {"number": 2, "name": "Structure name"}]}) |

**Table 1.** Different prompts for normal and thinking modes.

**References**

1.	Clinical Anatomy | Radiological Atlas. https://www.clinicalanatomy.ca/radiology.html. Accessed 11 Mar 2026
